# The immune modules conserved across the tree of life: Towards a definition of ancestral immunity

**DOI:** 10.1371/journal.pbio.3002717

**Published:** 2024-07-15

**Authors:** Aude Bernheim, Jean Cury, Enzo Z. Poirier

**Affiliations:** 1 Molecular Diversity of Microbes laboratory, Institut Pasteur, CNRS UMR3525, Paris, France; 2 Innate Immunity in Physiology and Cancer laboratory, Institut Curie, PSL Research University, INSERM U932, Paris, France

## Abstract

Immune defence mechanisms exist across the tree of life in such diversity that prokaryotic antiviral responses have historically been considered unrelated to eukaryotic immunity. Mechanisms of defence in divergent eukaryotes were similarly believed to be largely clade specific. However, recent data indicate that a subset of modules (domains and proteins) from prokaryote defence systems are conserved in eukaryotes and populate many stages of innate immune pathways. In this Essay, we propose the notion of ancestral immunity, which corresponds to the set of immune modules conserved between prokaryotes and eukaryotes. After offering a typology of ancestral immunity, we speculate on the selective pressures that could have led to the differential conservation of specific immune modules across domains of life. The exploration of ancestral immunity is in its infancy and appears full of promises to illuminate immune evolution, and also to identify and decipher immune mechanisms of economic, ecological, and therapeutic importance.

## Abstract 2

A subset of prokaryotic antiviral systems are conserved in eukaryotes and have crucial roles in immune pathways. This Essay introduces the concept of ancestral immunity, which refers to the set of immune modules (domains and proteins) conserved between prokaryotes and eukaryotes, discussing the topology of ancestral immunity and a putative evolutionary scenario for its existence.

## Introduction

Historically, immunology research has focused largely on animals and plants linked to therapeutic and biotechnological purposes. Complex immune strategies have been discovered in these organisms, including human innate immunity, during which pathogen infection results in the production of proinflammatory cytokines and the up-regulation of hundreds of anti-pathogen effectors termed interferon-stimulated genes (ISGs; Box 1) [1,2]. Plant and invertebrate immunity rely on different mechanisms such as the RNA interference (RNAi) pathway, which targets viruses in a sequence-specific manner [3]. These differences led to the widely accepted assumption that immune mechanisms would be clade specific, with little overlap between clades. As such, the immune defences of humans would be expected to be fundamentally different from those of *Arabidopsis thaliana*, as each is targeted by a different pool of pathogens. The constant arms race between pathogen and host indeed results in swift evolution of immune defence mechanisms tailored to a given pathogen or category of pathogens [4]. Under this assumption, defence mechanisms preventing bacteria from being infected by viruses (bacteriophages; also known as phages) would similarly be expected to be prokaryote specific. In line with this notion, 2 major antiphage defence mechanisms, restriction-modification enzymes and CRISPR-Cas enzymes, are absent in eukaryotes [5]. Recently, more than 150 antiphage systems with extremely diverse molecular mechanisms were identified [6]. Concomitant to the unravelling of bacterial immune defence was the realisation that some antiphage systems are in fact conserved in eukaryotes [7–9]. For example, cyclic GMP-AMP synthase (cGAS)-like enzymes, which produce cyclic dinucleotide second messengers upon pathogen detection, and viperins, which generate modified nucleotides blocking viral replication, protect bacteria and humans alike. Contrary to the conceptual framework of clade-specific immune mechanisms, a fraction of immune modules (domains or proteins involved in defence) are conserved between prokaryotes and eukaryotes.

**Box 1. Glossary**

*Italicised* words are defined within the glossary.

**Abortive infection** is the outcome of activation of numerous antiphage systems and results in the demise of infected bacteria, safeguarding the bacterial population by preventing generation of new virions. Abortive infection can be carried out by depletion of pivotal metabolites such as NAD+ (see *CBASS* and *Thoeris*) or by membrane permeabilization (see *Caspases and Gasdermins*).

**Ancestral immunity** refers to the immune modules (proteins and domains) conserved between certain prokaryotic and eukaryotic species. An ancestral immune module is not necessarily present in all prokaryotes and eukaryotes.

**Antiphage systems** are mechanisms of defence that protect bacteria from infection by viruses termed bacteriophages (also known as phages), either by intervening during the phage’s replication cycle, or by triggering the demise of infected bacteria (via *abortive infection*). See *CBASS*, and *Thoeris* for examples.

**Avs** are a family of antiphage systems regrouping different proteins that encode a signal transduction ATPases with numerous domains (STAND). Some Avs systems detect the terminase and portal proteins of phages. Avs may be at the evolutionary origin of *NLRs*.

**Caspases and gasdermins** are active in antiphage defence as well as in innate immunity of animals. Caspases are immune proteases that activate gasdermins by proteolytic cleavage. Cleaved gasdermins oligomerize into membranes, forming permeabilizing pores responsible for cell demise.

**CBASS/Pycsar** is an antiphage system that depends on CD-NTases—cGAS-like enzymes that detect phage infection. Detection leads to the production of a cyclic nucleotide second messenger that activates a downstream effector, inducing cell death. In certain cases, the effector is a STING protein fused to a *TIR domain*, which depletes cellular NAD+ upon activation (resulting in *abortive infection*). *cGAS* and *STING* are conserved in metazoans.

**cGAS/STING and cGLRs** refer to *innate immune* pathways of metazoans that detect bacterial and viral nucleic acids, leading to the production of inflammatory cytokines. In humans, cGAS detects cytosolic double-stranded DNA (e.g., from herperviruses) and produces the second messenger cGAMP, which signals to STING. cGAS-like receptors (cGLRs) are metazoan homologs of cGAS that can detect nucleic acid species other than DNA and produce various cyclic nucleotide second messengers. cGAS and STING originate from antiphage systems (see *CBASS/Pycsar*).

**CRISPR-Cas** is an antiphage system that protects bacteria by sequence-specific targeting of phage genomic material. Guide RNAs corresponding to phage sequences, encoded within the bacterial chromosome, guide the activity of the inhibitory Cas protein. CRISPR-Cas systems have been repurposed as a tool for genome editing.

**Detocs** (defensive two-component system) is a family of 3-gene defence systems. Upon phage recognition, Detocs degrades ATP, which can lead to premature phage lysis or *abortive infection*. The best-described Detocs system uses a PNP effector, which specifically cleaves ATP molecules into adenine and ribose-5′-triphosphate. PNP domains have been detected in eukaryotic proteins with immune-like architectures.

**Horizontal gene transfer** is the process by which 2 organisms exchange genetic material. Horizontal gene transfer is fundamental to prokaryotic biology but is less prominent in eukaryotes.

**Innate immunity** is one of the first lines of defence triggered by pathogen infection in *metazoans*. In humans, pathogen detection leads to the production of proinflammatory cytokines such as interleukin (IL)-1 and IL-18, as well as interferons. These mediators broadly signal to induce an anti-pathogen state within the host. Interferons trigger the production of hundreds of anti-pathogen effectors termed interferon-stimulated genes (*ISGs*). Cytokines also prompt the activation of adaptive immunity. Inflammation corresponds to the defensive state downstream of the stimulation of innate immunity, which can be abnormally activated in pathological conditions (autoimmunity).

**Interferon-stimulated genes (ISGs)** are anti-pathogen effectors transcriptionally up-regulated during the activation of *innate immunity* in metazoans; 300 to 400 ISGs restrict infection via various mechanisms (see *viperins*).

**Metazoans**. Group comprising all animals.

**NLRs** are pivotal proteins of *innate immunity* that detect pathogen infection as well as discontinuities of cellular physiology. Upon activation, they are involved in the formation of the inflammasome—a multiproteic platform dedicated to immune signalling. NLRs likely originate from antiphage systems such as *Avs*.

**piRNA pathway** is an animal mechanism of defence that protects the germline from the mobilisation of transposable elements. It relies on the Argonaute (Ago) family of proteins, which originate from antiphage systems and are also present in the *RNA interference* (RNAi) pathway.

**Restriction-modification** is an *antiphage system* that relies on the cleavage of phage genomes by a sequence-specific restriction enzyme. Bacterial DNA is protected from degradation by chemical modification. Restriction enzymes, widely used for experimental nucleic acid manipulation, originate from restriction-modification systems.

**Retrons** are antiphage systems composed of a reverse transcriptase, a noncoding RNA and an effector protein. Retrons generate DNA/RNA hybrids and function as tripartite toxin/antitoxin systems.

**RNA interference (RNAi)** is a mechanism of defence against viruses and transposable elements in plants, fungi, and animals. It relies on the activity of a Dicer protein, which recognises “foreign” nucleic acid and guides its degradation by the endonuclease Ago.

**SPARTA** is an antiphage system comprising a short prokaryotic Ago, with an APAZ domain fused to a *TIR domain*. SPARTA systems trigger cell death by depletion of NAD(P)+.

**Thoeris** is an antiphage system that depends on the detection of infection by ThsB, which bears a *TIR domain* that produces a second messenger, cADPR, from NAD+ degradation. cADPR activates ThsA, which leads to *abortive infection* through degradation of cellular NAD+.

**TIR domains** are domains present in antiphage systems, in which they participate in immune signal transduction (see *Thoeris*) or are responsible for *abortive infection* (see *SPARTA*). In animals, TIR domains of Toll-like receptors (TLRs) play a pivotal role in signal transduction via protein–protein interactions, leading to the activation of *innate immunity*.

**Viperins** are an antiphage system of bacteria; they are conserved across the tree of life and are present in humans as an *ISG*. Viperins produce modified nucleotides (ddh-nucleotides) that act as RNA chain terminators.

In this Essay, we propose the concept of ancestral immunity, which designates the pool of eukaryotic immune modules originating from prokaryotic immune defences. These modules are not necessarily present in all organisms but are detected across domains of life. We consider the following questions: what are the modules constituting ancestral immunity in terms of sequence, structure, and mechanism; how are these modules positioned in the tree of life; what are the selective pressures driving the existence of ancestral immunity; and why are some common building blocks of antiphage defence absent from ancestral immunity?

## The modules of ancestral immunity: What is conserved across domains of life?

**Fig 1:**
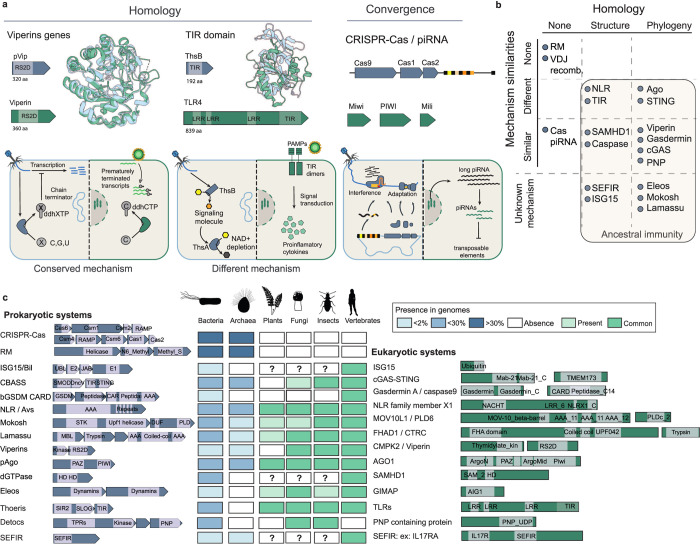
Defining ancestral immunity. ** (A)** Modules of ancestral immunity can be proteins, as exemplified by viperins, as well as domains, such as TIR domains, which are present in antiphage Thoeris and in mammalian TLRs. Eukaryotes also encode defences that are unrelated to those of bacteria, the mechanisms of which can be analogous to prokaryotic defence systems. For example, CRISPR-Cas of bacteria and the piRNA pathway of animals are 2 evolutionarily unrelated mechanisms of nucleic acid-guided memory [10]. Top panels: examples of prokaryotic (blue) and eukaryotic (green) proteins of interest. Structural comparison for viperins and TIR domains (generated with AlphaFold). Bottom panels summarise the modules’ mechanisms of action; prokaryotes are labelled in blue and eukaryotes in green. **(B)** Proposed classification for the modules of ancestral immunity according to shared ancestry (homology) and mechanism of action. VDJ recombination is an innovation of jawed vertebrates absent in bacteria, with no shared ancestry nor mechanistic conservation. Viperins display shared ancestry and mechanistic conservation. The boundaries of ancestral immunity are highlighted. Unknown mechanism refers to an undetermined molecular mechanism in prokaryotes and/or eukaryotes, despite a known (experimentally assessed) function in immunity. **(C)** Conservation of the modules of ancestral immunity in different clades of eukaryotes and prokaryotes. Prokaryotes: the percentage of genomes in which the module is detected are represented by shades of blues (based on the DefenseFinder database [11]). Eukaryotes: as inferred from the literature, common (widely conserved), present (detected in some genomes), or absent. “?” indicates that the literature is insufficient to assess conservation. TIR, Toll-interleukin-1 receptor; TLR, Toll-like receptor.

The term “conservation” requires scrutiny before applying it to the description of ancestral immunity. In the relevant literature, conservation is used to describe both shared ancestry (an evolutionary relationship between 2 proteins as captured by sequence and/or structural homology) and shared mechanisms, which can be performed by homologs or by evolutionarily unrelated proteins (Fig 1). These 2 dimensions may or may not overlap (Fig 1B). Proteins that likely share common ancestry (with sequence and/or structural homology) can function through different mechanisms while immune mechanisms conserved across domains of life, such as nucleic acid-guided memory, could be performed by unrelated pathways, representing evolutionary convergence [5]. We focus here on modules that are conserved in the sense of shared ancestry.

### A typology of ancestral immunity

Antiphage systems can be composed of a single protein or multiple proteins that act in concert, with each protein bearing one or several domains [6]. Recent work demonstrated that certain antiphage modules are conserved in eukaryotes and play a role in immune defence [12–28]. At least 10 examples of immune modules (i.e., protein domains or full proteins) shared by bacteria and eukaryotes have been documented (Fig 1C) [7–9].

Conservation of immune proteins across domains of life is perhaps best embodied by viperins, which are highly conserved at the structural and amino acid sequence levels across a range of bacterial, archaeal, and eukaryotic organisms [29–31]. These enzymes display a remarkably high degree of sequence similarity, with 42% shared identity between human viperin and its closest bacterial counterpart [12,32]. Bacterial viperin blocks phage infection by producing modified nucleotides that act as RNA chain terminators. The human viperin is an ISG that thwarts infection by a range of viruses through a similar mechanism. In mammals, other antiviral effectors such as GIMAPs and SAMHD1 similarly seem to originate from antiphage systems, albeit with less stringent conservation [24,33].

The Toll-interleukin-1 receptor (TIR) homology domain serves as an illustration of functional diversity from a common ancestor. TIR domains can function as antiphage effectors in bacteria, including in proteins of the Pycsar and SPARTA antiphage systems, by degrading NAD+ and causing cell death via an abortive infection mechanism [20,23]. In the bacterial Thoeris system, the TIR domain of the protein ThsB produces a second messenger derived from NAD+ processing that activates the antiphage response [26]. In plants, TIR domains perform immune signal transduction through their enzymatic activity [34]. In mammals, TIR domains constitute the cytosolic portion of Toll-like receptors (TLRs)—a group of pattern recognition receptors that detect pathogen infection [2]. TIRs of animal TLRs are devoid of enzymatic activity and rely instead on protein–protein interactions for signal transduction, inducing proinflammatory cytokine production. The TIR example demonstrates that a conserved domain can perform different biochemical actions within immune pathways.

The presence of conserved domains in different eukaryotic phyla does not necessarily imply that they originate from a common ancestor. Nucleotide-binding oligomerization domain, leucine-rich repeat-containing proteins (NLRs) are present in proteins of the Avs antiphage system, as well as in fungal immune actors and mammalian inflammasome proteins, and are responsible for inducible cell death downstream of infection [13,35,36]. However, NLRs evolved independently in plants and animals [37].

Antiphage CBASS/Pycsar systems comprise a cGAS-like nucleotidyltransferase enzyme that produces a cyclic di-nucleotide second messenger upon phage infection, which is responsible for the activation of an antiviral effector [20,25]. The nature of the effector is operon specific and can include a STING protein fused to a TIR domain [19]. cGAS and STING are conserved in metazoans and play a pivotal role in the activation of innate immunity upon DNA virus infection in humans [17,18,21,28]. In contrast to its role in bacteria, in humans STING is used as a signal transduction platform, inducing the production of proinflammatory cytokines [38].

Pathogen recognition in eukaryotes also results in cell demise, orchestrated by caspases and gasdermins [39]. Activated caspases cleave gasdermins, giving rise to pore-forming proteins that permeabilise the plasma membrane. There is sequence and structure conservation of caspases and gasdermins, respectively, indicating that such a cell death mechanism originates from bacteria [16,40].

The functional linkage of several ancestral immune modules within 1 pathway does not seem to be explained by bulk inheritance of all modules populating the pathway, but rather by convergent evolution, as observed with cGAS and STING, which were independently inherited in eukaryotes [29]. The activity of viperins depends on the synergistic action of the kinase CMPK2. In bacteria, 15% of genes encoding viperins are located next to genes encoding a kinase similar to CMPK2. Although human viperin seemingly emerged from an archaeal ancestor, CMPK2 is thought to have an independent bacterial origin [12,29,31]. Inheritance of multiple proteins of an antiphage system may resemble bulk pathway inheritance but could originate from separate events of protein acquisition followed by convergence in immune pathway assembly.

A conserved module can have an identical mechanism of action (e.g., viperins) or a host-specific mechanism (e.g., enzymatic TIRs in bacteria and plants and non-enzymatic TIRs of mammalian TLRs; Fig 1). By contrast, evolutionary convergence exists between unrelated pathways (e.g., nucleic acid-guided memory in bacteria and animals performed by CRISPR-Cas and the piRNA pathway, respectively) as do non-conserved mechanisms (restriction-modification specific to bacteria; VDJ recombination specific to jawed vertebrates) [10]. Such classification is, however, a matter of discussion. The hundreds of millions of years of evolution separating the bacterial, archaeal, and eukaryotic kingdoms led to significant divergence in sequence, which often makes the unambiguous attribution of common origin almost impossible. A rigorous definition of mechanistic conservation can also be debated: for example, should the module’s biochemistry be identical; and should the modules be triggered by a similar signal?

Conservation of antiphage modules in eukaryotic immunity is characteristic of tinkering evolution, with a combinatorial use of modules to build tailored pathways, as well as modifications of the modules’ properties adapted to specific host contexts.

### What are the boundaries of ancestral immunity?

Belonging to ancestral immunity requires a module to perform an immune function in the prokaryotic and eukaryotic superkingdoms, with or without conservation of biochemical and molecular mechanisms. The extent to which a given module of ancestral immunity is conserved across the tree of life (i.e., the number of eukaryotic and prokaryotic species encoding at least 1 likely homolog) is highly variable (Fig 1). Viperins are present in most clades of eukaryotes and prokaryotes but absent in plants [12,29]. cGAS is largely conserved in metazoans, with homologs forming a family of cGAS-like receptors (cGLRs) [18,24]. The establishment and interpretation of such data is hindered by current capacity to detect homologs of these pathways across long evolutionary distances and the limited number of available diverse eukaryotic genomes. The precise delineation of families of immune modules will require experimental validation and mechanistic studies. Nonetheless, focusing on well-established examples (in which homology is reliably detected) indicates a patchy distribution of ancestral immune modules. Interestingly, the most common systems in bacteria and archaea—CRISPR-Cas and restriction modification, which are present in more than 40% and 80% of prokaryotic genomes, respectively—are absent in eukaryotes (Fig 1C) [11]. Conversely, some very rare immune modules in bacteria, such as viperins (which are present in less than 2% of bacterial genomes), are present in eukaryotic genomes. This overall patchy distribution across the tree of life suggests specific selective pressures.

Eukaryotes have been subject to numerous bursts of innovation, which gave rise to novel cellular biology (e.g., introns and genome duplications) as well as to original immune mechanisms unrelated to bacteria. For example, VDJ recombination, which gives rise to the B and T cells of mammalian adaptive immunity, originates from co-option of a RAG-like transposable element protein [41]. Yet, eukaryotes also reused antiphage modules, which play pivotal roles in anti-pathogen defence across domains of life. We therefore ask: what could be the evolutionary forces driving the emergence of ancestral immune modules?

## Evolutionary drivers behind ancestral immunity

Conservation of antiphage homologs in eukaryotes could be explained by the existence of common selective pressures to build an efficient immune response, related to the inevitable requirements of functioning pathways (constraints) as well as to the need for constant evolution to face pathogens (opportunities).

### Common evolutionary constraints on immunity

Bacteria and eukaryotes face similar constraints related to the implementation of defence systems, which introduce selective pressures likely contributing to the selection of ancestral immunity. First, both bacteria and eukaryotes require specificity in pathogen detection. The response is triggered by specific cues of pathogen or cellular origin that introduce a discontinuity within cellular physiology. This corresponds to a rapid change in the molecules specifically surveyed by immune defence [42,43]. For example, increased cytosolic double-stranded DNA concentration in human cells is detected by the sensor cGAS [38]. The effector response targeting the pathogen necessitates a similar level of specificity, with the exception of mechanisms of inducible cell death. Second, temporality is important. The immune response must be swiftly triggered, but controlled in time and return to physiological levels. Finally, orthogonality is needed. The cross-talks between immune defence and other cellular pathways (e.g., metabolism and cell division) require tight control to avoid perturbations of nonimmune pathways and prevent unwanted activation of an immune response. For example, upon DNA detection, human cGAS produces a specific second messenger, 2′3′-cGAMP, which signals to STING, but generally not to other proteins. 2′3′-cGAMP is not generated by other, nonimmune enzymatic reactions [38].

It is likely that such rigid requirements can be fulfilled by only a limited number of modules. Antiphage proteins represent a formidable bucket of solutions from which to draw, because the selective pressures stemming from the aforementioned constraints shaped antiphage systems. Conservation of prokaryotic modules is also possible because bacteria and eukaryotes share a grammar of cellular constituents (e.g., nucleic acids and central metabolites such as NAD+) that can be harnessed and/or targeted for immunity.

### Common evolutionary opportunities: The power of tinkering

The presence of an efficient immune response imposes a selective pressure on the pathogen to diversify, thereby constraining host immunity to catch up, resulting in an arms race between pathogen and host (commonly known as the Red Queen Hypothesis [4]). Across the tree of life, the ability to evolve and adapt is therefore essential for immune defences. In addition to mutation, recombination is a powerful evolutionary force for antiphage systems, which demonstrate a combinatorial organisation of proteins and domains. Various antiviral effectors can pair up with a given viral receptor, generating different “flavours” of the same antiphage system [23]. Similarly, a given antiphage domain can participate in several antiphage systems. Lamassu displays 10 different effectors, 6 of which participate in other antiphage systems such as CBASS or retrons [23]. Selective pressures stemming from host–pathogen coevolution thus result in the evolution of antiphage domains that can be swiftly tinkered with to face a given threat.

Another prediction of the Red Queen Hypothesis is that antiphage systems could have been selected for their evolvability (i.e., their capacity to mutate and change). Although such a claim is not backed up by data, it is striking that conserved immune modules use clade-specific biochemistry within the boundaries of a shared mechanism of action. For example, prokaryotic viperins produce several types of modified nucleotides while eukaryotic viperins are limited to either CTP or UTP derivatives [12,31]. Similarly, cGLRs and bacterial cGAS produce a range of cyclic nucleotide signalling molecules, indicating that modules can use diverse biochemistry without losing ancestral functions of specificity and orthogonality [18]. Highly tinkerable and evolvable modules may have been selected in diverse domains of life, especially in the context of adaptation to eukaryotic-specific pathogens (which comprise a higher number of RNA viruses [44]).

The aforementioned selective pressures may explain the existence of ancestral immunity. Yet, certain antiphage systems are strikingly absent from the list. Although restriction-modification is the most abundant bacterial defence system, restriction-modification enzymes have never been documented in eukaryotes (apart from an anecdotal occurrence [45]), and neither has CRISPR-Cas. By contrast, rare systems such as gasdermins, which are encoded by 0.5% of prokaryotic genomes, are conserved in a large fraction of eukaryotes. What could be the rules governing the conservation of a subset of antiphage systems in eukaryotes?

### The impact of a transmission bottleneck on the composition of ancestral immunity

The differential conservation of antiphage modules in eukaryotes could be explained by a relative enrichment or loss of specific modules in prokaryotic precursors of eukaryotes, as suggested by Wein and Sorek [7]. This scenario implies that a limited number of modules were transmitted to the last eukaryotic common ancestor (LECA), essentially constraining the diversity of modules available for eukaryotic evolution. Eukaryotes presumably originate from an ancestral archaean most closely related to present day Asgard archaea [46]. Hypothesising that the composition of defence systems in eukaryotic ancestors explains the differential conservation of modules in modern-day eukaryotes implies that modules present in Asgard archaea and eukaryotes should be relatively similar. Analysing hundreds of Asgard genomes reveals that antiviral systems of Asgard archaea resemble those of other archaea and bacteria, with the widespread presence of systems absent in eukaryotes, such as restriction-modification and CRISPR-Cas. Some immune modules present in eukaryotes are indeed prevalent in Asgard archaea, such as prokaryotic Agos and viperins. Genomic and phylogenetic analyses indicate that prokaryotic Agos and viperins proteins were likely present in LECA and vertically inherited [29,31,47]. Conversely, ancestral immune modules of bacteria and eukaryotes, such as Detocs/PNP and gasdermins, are absent from archaeal genomes and were likely acquired later during evolution through horizontal gene transfer between bacteria and ancestral eukaryotes [29,31,47].

If transmission from LECA could account for the conservation of a subset of immune modules in eukaryotes (e.g., Agos and viperins), such a scenario fails to explain the presence (e.g., PNP-containing proteins and gasdermins) or absence (e.g., restriction-modification and CRISPR-Cas) of a significant subset of modules. The transmission bottleneck linked to the appearance of LECA does not entirely explain the composition of ancestral immunity, especially when considering that ancestral immune modules could have been later acquired by horizontal gene transfer. This suggests that additional pressures exist to explain the (counter-)selection of a given antiphage module in eukaryotes.

## Selective pressures driving the composition of ancestral immunity

### Immune molecular mechanisms relevant to diverse pathogens

A conservative hypothesis justifying the existence of ancestral immunity across domains of life would be a continuous selective pressure exerted by a given pathogen. Herpesviruses, which infect eukaryotes as diverse as marine protists and humans, represent an interesting candidate, as multiple human homologs of antiphage modules target herpesviruses [24,35]. NLR-related modules target phages and human herpesviruses, while bacteria and human Lamassu thwart phage and herpes simplex virus 1 infection, respectively. The phage order *Caudovirales* and eukaryotic herpesviruses are phylogenetically related, and regrouped in the superfamily of *Duplodnaviridae*, characterised by structural conservation of several viral proteins, including the HK97 fold of the major capsid as well as the portal and terminase proteins [48,49]. Herpes-like pathogens were even suggested to have targeted LECA [50]. It is tempting to speculate that certain antiphage systems were conserved throughout eukaryotic evolution to respond to the constant pressure of herpes-like viruses.

Another key determinant of the conservation of immune modules may relate to the ability of pathogens to thwart antiviral pressure through mutation. Indeed, the selective pressure exerted by an efficient mechanism of defence pushes for the selection of pathogen variants that evade recognition or antiviral effectors (as per the Red Queen Hypothesis). The ability to mutate while maintaining function depends on the structure and function of viral proteins. For example, polymerases that replicate viral genomes during infection are considered more constrained (i.e., less tolerant to mutation than other proteins). Defence modules targeting constrained viral elements thus provide a selective advantage and may have therefore been conserved. Recent work demonstrated the ability of an NLR-related antiphage system Avs to be triggered by the portal proteins of human herpesviruses in addition to those of phages. Avs recognises portal proteins from diverse phages that have <5% sequence similarity but that have conserved ancestral structures [35]. Similarly, viperins produce modified nucleotides that interrupt viral genome elongation during replication [12,32]. If modified nucleotides are incorporated by DNA-dependent RNA polymerases from phages, they are similarly used by RNA-dependent RNA polymerases of eukaryotic RNA viruses. The production of modified nucleotides disabling viral genomes appears to represent a broad antiviral strategy that acts against unrelated viral families. Such an efficient molecular mechanism could have been under constant selective pressure to be maintained, even with the evolution of novel viral families.

### Cost–benefit trade-offs of antiviral mechanisms

The use of antiviral mechanisms is associated with significant energy costs as well as potential toxicities linked to autoimmunity. For example, in bacteria, CRISPR-Cas systems can mistakenly target the host genome, resulting in cell death [51]. Patients with Aicardi–Goutières syndrome have elevated levels of inflammation at baseline, with severe cases displaying reduced life expectancy [52]. The selection or counter-selection of a given mechanism needs to be understood as a trade-off, in which the benefits of protection must outweigh the inherent cost of implementing an active mechanism. Host-specific variations in the cost–benefit balance could explain the differential conservation of antiphage systems. For example, mechanisms may be counter-selected due to their incompatibility with cellular processes unrelated to immunity. In bacteria, CRISPR-Cas systems can compete with DNA repair pathways, particularly non-homologous end joining, in the context of horizontal gene transfer, resulting in counter-selection of the CRISPR-Cas system in specific genetic backgrounds [53]. The larger size of eukaryotic genomes (10^6^ for the bacterial chromosome versus the 10^9^ nucleotides of human DNA) increases the probability of “accidental” CRISPR or restriction-modification-mediated restriction, leading to autoimmunity. In eukaryotes, regulation of gene expression relies heavily on DNA methylation, which is also how bacterial genomes avoid being targeted by restriction-modification systems. Use of DNA methylation as a means of gene regulation is thus at odds with its use within a restriction-modification system. Conversely, the existence of host-specific processes can influence the cost–benefit balance. Restriction-modification enzymes and CRISPR-Cas systems not only act as antiphage systems but also as regulators of genetic material obtained through horizontal gene transfer—a key beneficial function. In eukaryotes, horizontal gene transfer is less extensive, reducing the benefits provided by such systems. This could tilt the cost–benefit balance, leading to counter-selection. Another hypothesis that is not mutually exclusive relates to the appearance of the eukaryotic nucleus. Genome replication of jumbo phages occurs within nuclei-like structures that physically shield DNA from restriction-modification and CRISPR-Cas [54,55]. The eukaryotic nucleus may have had a profound impact on the cost–benefit trade-off of restriction-modification and CRISPR-Cas, rendering both systems obsolete.

## Putative scenario to explain the evolution of ancestral immunity

**Fig 2:**
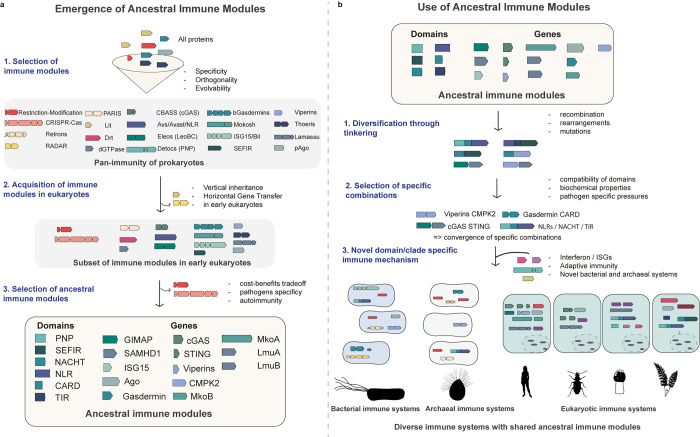
Putative scenario to explain the evolution of ancestral immunity. **(A)** The emergence of ancestral immune modules depends on the selection of immune modules within prokaryotes, subject to selective pressures (step 1). A subset of modules is acquired by eukaryotes through vertical inheritance and horizontal gene transfer (step 2). Modules are selected within a eukaryotic framework (step 3). **(B)** Acquired immune modules are then utilised (through selection) for the construction of immune pathways, with diversification through tinkering (step 1), and selection of a subset of “tinkered-with” modules (step 2), which are included in and completed by original, nonbacterial immune mechanisms (step 3).

Based on the considerations discussed above, we propose a putative evolutionary scenario explaining the emergence of ancestral immunity (Fig 2A) as well as the use of ancestral immune modules in the construction of diverse immune systems across the tree of life (Fig 2B).

### Emergence of ancestral immunity

In the **first step**, prokaryotic immune modules are selected on the aforementioned constraints (specificity, temporality, and orthogonality), resulting in a diverse set of modules of defence, some of which are clade or species specific (Fig 2A). These modules constitute the pan-immunity of prokaryotes. During the **second step**, a subset of these modules is transmitted to eukaryotes. Module transmission to eukaryotes could have been partly contingent on the types of prokaryotic immune modules present at that time (i.e., a transmission bottleneck during the appearance of LECA). Rather than being singular, we speculate the existence of multiple events of transmission, over a restricted but fairly long period running from early eukaryogenesis to the emergence of complex multicellularity. This hypothesis also implies that certain modules of prokaryotic immunity were never acquired by eukaryotes, with no possibility of ever becoming ancestral immune modules. In the **third step**, the subset of modules transmitted to eukaryotes is subjected to selection. The emergence of eukaryotic specificities leads to cost–benefit trade-offs that apply to each module, sometimes driving counter-selection and loss (e.g., restriction-modification and CRISPR-Cas). The combination of an acquisition bottleneck (second step) followed by eukaryote-specific selective pressures (third step) likely led to the emergence of a defined set of ancestral immune modules.

### From ancestral immune modules to diverse immune systems

In the **first step** of diversity generation, ancestral immune modules are combined through tinkering mechanisms such as recombination (e.g., NLRs) and mutations (e.g., TIR domains; Fig 2B). Tinkering can operate at the level of a single gene, which may conglomerate several ancestral immune domains acquired separately, as well as at the pathway level, through the association of genes (e.g., cGAS-like receptor and STING; viperins and CMPK2). In the **second step**, diverse combinations resulting from tinkering are subject to selection, including from pathogens. An important parameter may be the compatibility of domains or proteins. One may expect, for example, that an enzyme producing a nucleotide-derived second messenger (e.g., a cGAS-like enzyme) may function more efficiently, and be positively selected, when associated with a protein capable of specifically interacting with that sort of second messenger (e.g., STING). Other parameters would be under selection, such as biochemical properties and the ability to be included in (or excluded from) a given pathway. In parallel, novel immune defences, unrelated to ancestral immunity, appear in eukaryotes, resulting in the currently known landscape of immune mechanisms.

### Considerations of the putative evolutionary scenario

Following Gould’s famous thought experiment “replaying life’s tape,” we could ask whether rerunning eukaryotic immune evolution would lead to an identical result [56]. Would ancestral immunity be composed of a different set of modules? Would it exist at all? We propose that ancestral immunity is a product of both contingency and determinism. If ancestral immunity drew from the bucket of prokaryotic modules, it was contingent on the type of modules available for transmission at that time. Selective pressures then determined the conservation or loss, as well as the variations, of ancestral immune modules.

## Conclusions and perspectives

### Towards a definition of ancestral immunity

Antiphage modules are not only conserved in eukaryotes, but populate many stages of innate immune pathways. Eukaryotic immunity appears to emerge in part from the selection of diverse antiphage modules. This does not preclude major innovations unrelated to bacteria, but rather suggests that antiphage modules may have been the first evolutionary bricks used in the construction of immunity. We propose the concept of ancestral immunity, which designates the pool of eukaryotic immune modules originating from prokaryotic immune defences.

### The benefits of studying ancestral immunity

Identifying the modules of ancestral immunity can illuminate the immunological organisation of prokaryotes and eukaryotes. The vast body of literature on mammalian immunology can be exploited to understand antiphage immunity. Gasdermins belong to ancestral immunity and trigger cell demise upon infection. By forming pores in the plasma membrane, human gasdermins also allow the release of the proinflammatory cytokines interleukin (IL)-1 and IL-18 [39]. Could bacterial gasdermins act in a similar manner, liberating signalling molecules that act on neighbouring bacteria?

Conservation of antiphage systems can be used to discover novel immune components in eukaryotes. A recent proof-of-principle study identified several novel human antiviral effectors belonging to ancestral immunity [24]. This approach can be applied to any eukaryote of interest and could prove particularly useful to organisms less amenable to experimentation.

Understanding the variations of ancestral immune modules across related species (mutations, gains, or losses) as well as the acquisition of immune actors outside of ancestral immunity may enable a better understanding of the evolution of immunity. Indeed, the study of clade- and potentially species-specific immune defences has recently been coined evo-immuno, by analogy with the evo-devo discipline [57]. Are the modules of ancestral immunity preferential providers of diversity? Could certain modules of ancestral immunity be replaced by eukaryotic innovation?

Ancestral immune modules are by definition conserved, while performing an immune function that seemingly requires hypervariability to adapt to pathogens in constant evolution. The molecular means by which this paradox is solved have begun to be unravelled [31].

With the existence of conserved modules comes the evolution of immune blockers by pathogens. Phages have developed myriad proteins dedicated to thwarting antiphage systems, including sponges that sequester cyclic di-nucleotide messengers [58]. Could different viruses infecting bacteria and humans encode similar anti-defence proteins to block ancestral immune modules? There is structural similarity between viral proteins dedicated to counter-defence, exemplified by Acb1 of phages and ligT-like phosphodiesterases of poxviruses, which target cGAS second messengers [59–61]. Could certain viruses that infect hosts from different kingdoms encode blockers of ancestral immune modules effective across domains of life [62]? Surveying ancestral immunity from a pathogen perspective may enable the discovery of novel viral strategies targeting immunity.

### Future directions

Numerous tasks lie ahead to give substance to the concept of ancestral immunity. Modules belonging to ancestral immunity need to be catalogued, with the help of sequence and structural clustering [63]. The distribution and evolutionary history of these modules need to be unravelled across the tree of life, as do their molecular mechanisms of action and species-specific variations.

The study of ancestral immunity aims at understanding the general rules of immunology, complementing the limited examples extracted from model organisms. It will improve comprehension of non-model organisms, including species of economic, cultural, and environmental importance. Manipulation of immune activation, including inflammation driven by innate immunity, is a well-documented therapeutic strategy. In patients with cancer, an inflamed microenvironment improves the efficiency of antitumor immunity, while dampening inflammation enables control of certain autoimmune diseases. We foresee that the exploration of ancestral immunity will open new avenues of research in human health.

## Abbreviations

cGAS: cyclic GMP-AMP synthase
ISG: interferon-stimulated gene
LECA: last eukaryotic common ancestor
NLR: nucleotide-binding oligomerization domain, leucine-rich repeat-containing protein
RNAi: RNA interference
TIR: Toll-interleukin-1 receptor
TLR: Toll-like receptor
